# Consumers’ Use of Web-Based Information and Their Decisions About Multiplex Genetic Susceptibility Testing

**DOI:** 10.2196/jmir.1587

**Published:** 2010-09-29

**Authors:** Kimberly A Kaphingst, Colleen M McBride, Christopher Wade, Sharon Hensley Alford, Lawrence C Brody, Andreas D Baxevanis

**Affiliations:** ^3^Department of Biostatistics and Research EpidemiologyHenry Ford HospitalDetroit, MIUSA; ^2^Genome Technology BranchNational Human Genome Research InstituteNational Institutes of HealthBethesda, MDUSA; ^1^Social and Behavioral Research BranchNational Human Genome Research InstituteNational Institutes of HealthBethesda MDUSA

**Keywords:** Genetic testing/methods, genetic testing/psychology, genetic predisposition to disease/psychology, health knowledge, attitudes, practice, health surveys, internet/utilization, polymorphism, single nucleotide, public health/methods, risk assessment/methods

## Abstract

**Background:**

Few data exist to inform concerns raised by online direct-to-consumer marketing of genetic susceptibility tests, such as those offered by  commercial entities like 23andme, Navigenics, and DNA Direct. The Multiplex Initiative, a population-based study of healthy adults, provides the first opportunity to evaluate how use of a Web-based decision tool that conveyed information about a genetic susceptibility test influenced individuals’ test decisions.

**Objective:**

To inform the ongoing debate over whether individuals offered genetic susceptibility testing without the involvement of a health care provider (eg, through direct-to-consumer testing) can make informed decisions about testing when guided by online decision aids.

**Methods:**

Participants were 526 members of a large health maintenance organization aged 25 to 40 years old who visited a study website. Multivariate logistic regression models were tested to examine the association of website usage with downstream test decisions.

**Results:**

Participants viewed an average of 2.9 of the 4 pages introducing the multiplex test, 2.2 of the 8 pages describing the health conditions, and 3.2 of the 15 pages describing the genes. For each page viewed, participants were more likely to describe their decision-making as easy (odds ratio [OR] 1.04, 95% confidence interval [CI] 1.01-1.07) and to decide to be tested (OR 1.08, 95% CI 1.05-1.11).

**Conclusions:**

Healthy adults in this study perceived Web-based genomic information presented using evidence-based communications approaches to be helpful in supporting both decisions to test and not to test. Continued research is needed to ensure that these results generalize to target groups with lower literacy and less Internet savvy.

## Introduction

Several new genetic tests provide individuals with information about their susceptibilities to a wide array of common health conditions. The availability of these tests is expected to increase greatly over the next decade as more gene-disease associations are identified [1]. Despite the lack of data on clinical effectiveness, commercial entities are even now marketing such tests online directly to consumers (eg, 23andme, Navigenics, and DNA Direct), a practice growing in prominence in the United States [1-4]. This report presents data from the Multiplex Initiative [5,6] to inform the ongoing debate over whether individuals offered genetic susceptibility testing without the involvement of a health care provider—that is, direct-to-consumer (DTC)—can make informed decisions about testing when guided by online decision aids.

One of the most hotly contested issues has been focused on the challenges of communicating complex information about genetic risk for common, chronic diseases [7,8]. Given the complex etiology of common diseases, critics have expressed concern that the public will not understand genomic information without the assistance of a health care provider and will be unable to make informed decisions about taking genetic susceptibility tests [7,9,10]. Of particular concern is whether individuals can understand the limitations of the information generated by these tests and appreciate what cannot be learned from such tests [7].

Direct-to-consumer companies’ reliance on interactive Web-based approaches adds complexity to these communication issues [11,12]. The Internet increases dissemination potential, thereby enabling reach to ever-increasing proportions of the population with health information and genetic testing [5,13,14]. However, Web-based approaches also may be inadequate for communicating complex genetic susceptibility information, particularly for individuals with limited computer or health literacy skills, when compared with interpersonal approaches [15,16,17]. Indeed, existing DTC marketing websites have been shown to use language that is too difficult for most of the US public to comprehend and to have limited content in areas that may be critical for decision-making [18].

Most prior research regarding comprehension and uptake of genetic testing has occurred in the context of high-risk familial cancer syndromes. The majority of those who present for such testing have already decided to be tested [19,20]. These studies, therefore, have provided little insight into differences among those who decide to be tested and those who considered testing but decided not to test or into whether online information can support such decision-making. To date, there have been no population-based studies evaluating whether or not individuals can use information made available online to make an informed decision about testing. These questions can be examined using data generated by the Multiplex Initiative [1].

The Multiplex Initiative was designed to develop the infrastructure needed to evaluate a multiplex test (ie, a test that includes multiple genetic variants for multiple health conditions) taken by healthy adults insured through a large managed care organization. The study provided the first opportunity to systematically present genetic susceptibility information based upon best communication practices and then to examine individuals’ responses to the information. Despite the fact that testing was offered at no cost and the target population was insured, we reported previously that those who logged on to a study website to consider testing and subsequently elected to be tested were significantly more likely to be college educated and white than those who did not log on or were not interested in testing [6].

In this report, we pose three specific research questions: (1) How do participants in the Multiplex Initiative engage with different content areas of information provided on the website? (2) How do participants rate the quality and usefulness of the website information? (3) Is website use associated with decisions about genetic testing?

## Methods

### Study Design and Participants

The Multiplex Initiative has previously been described in detail [6]. In brief, study participants were selected from a pool of 350,000 members of a large Midwestern health maintenance organization. Selection criteria included being between the ages of 25 and 40 years, having been enrolled in the plan for at least two years, and not having any of the health conditions included on the Multiplex test. Groups traditionally underrepresented in genetics research (ie, men, blacks, and those with lower education) were oversampled as described in detail elsewhere [6]. All procedures were approved by the institutional review boards of the National Human Genome Research Institute and the Henry Ford Health System.

A baseline telephone assessment was attempted with 6348 sampled individuals. Of these, 1930 completed the assessment and were invited to visit the study website. Individuals completed a consent process as part of the initial Web module. Participants were told that they would be asked to complete brief questionnaires and to review Web content. A total of 612 individuals visited the website, and 527 completed all four website-based assessments. Although website visitors who did not complete all four assessments were similar to those who completed all assessments based on age, gender, educational attainment, and marital status, white participants were more likely to complete all assessments than black participants (*P* = .002). Following completion of the website portion of the study, interested individuals completed an in person clinic visit, and then a blood draw was performed on those individuals who decided to undergo testing. The analyses presented here are based on 526 individuals who visited the website, completed all four website assessments, and for whom data regarding the testing decision were available. These 526 individuals had a mean age of 34.6 years. Half (263/526) were white. A majority were female (297/526 or 56.5%), and most were married or in a partnered relationship (336/526 or 63.9%).

### Website Content

The content of the Multiplex Initiative study website was developed by an interdisciplinary team of researchers, drawing on prior research and best practices in health literacy and risk communication. Health literacy principles were used to develop the information content of the website. For example, the scope of the content was limited to what the team considered the most essential information needed to support participant decision-making [21]. In addition, the information was organized using a layered approach [21,22]. Participants were offered a menu of content topics and could then choose the order and amount of content reviewed, allowing those participants who wanted more detailed content to find that information. We avoided using technical jargon where possible (eg, using “risk version” instead of “risk-increasing gene variant”) and defined jargon where it was used (eg, “a risk factor is anything that increases your chance of getting a health condition”). We drew upon prior risk communication research to convey risk information on the website. For example, risk estimates were given using an “n in 100” format, which prior research has shown to convey risk information best to lay individuals [23,24]. In addition, we selected pictograph graphics to visually convey the risk information, a type of graphic that has been shown to convey this information to lay audiences more effectively than alternative graphic formats [24].

**Figure 1:**
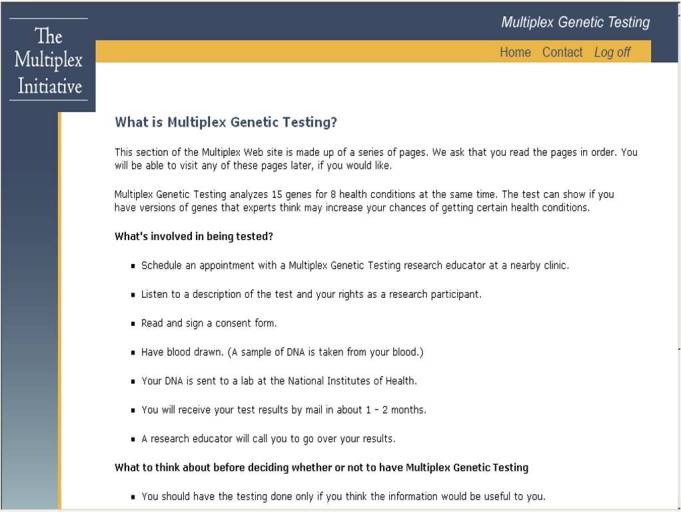
Example website page providing information about Multiplex Genetic Testing

**Figure 2:**
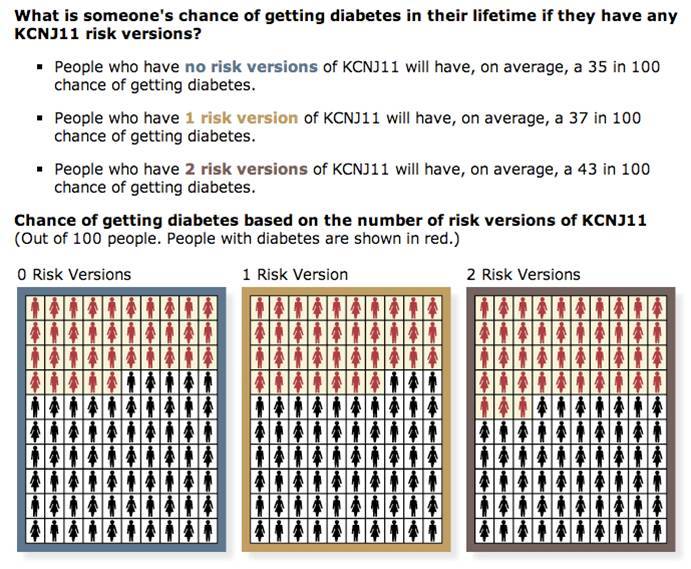
Example website page providing information about a gene on the test

The website content was organized into four modules: (1) Multiplex Genetic Testing: What it Can and Cannot Tell You; (2) Diseases and Genes on the Multiplex Genetic Test; (3) Your Rights if You Take Part in Multiplex Genetic Research; and (4) Your Decision to be Tested or Not (see Textbox 1). Two examples of website pages are shown in Figures 1 and 2. The participants received small incentives (gift cards up to US $50 from a national retail chain) for completing the website assessments.

**Study website content and assessment points**

**Module 1: Multiplex Genetic Testing: What it Can and Cannot Tell You (4 pages)**

 

*Content topics*

 

- Definition of multiplex genetic testing
- Testing procedures
- Overview of health conditions and genes
- Meaning of “genetic risk”
- Importance of health habits and other factors in disease risk

**Module 2: Diseases and Genes on the Multiplex Genetic Test (23 pages)**

 

*Content topics*

 

For the eight health conditions:

- Description of condition
- Known risk factors for condition
- Genes that affect risk of condition

For the 15 genes:

- Brief description of gene action
- Increased risk associated with gene variant
- Prevalence of risk-increasing gene variant in population
- Limitations of what is known about gene-disease association
- Scientific references

*Assessment used in analysis*

 

· Perceptions of website information in Module 2

**Module 3: Your Rights if You Take Part in Multiplex Genetic Research (4 pages)**

 

*Content topics*

 

- Researcher responsibilities
- Rights of research participants
- Test procedures

**Module 4: Your Decision to be Tested or Not (1 page)**

 

*Assessments used in analysis*

 

- Ease or difficulty of decision making
- Interest in making clinic appointment

### Website Tracking

Each study participant was given a unique log-in ID/password pair that enabled their visit to the Multiplex website to be tracked. Each time a page on the website was accessed, a tracking database stored the participant’s unique log-in ID, the session number for that user (ie, whether this was their first, second, or subsequent visit to the Multiplex website), and the date and time that the page was requested. Log-in IDs were randomly generated and created so that the responses of a particular individual could not be identified.

### Measures

The measures included in the report were collected at three time points: baseline telephone assessment, website assessments (about 1 to 2 weeks after baseline), and whether individuals attended the clinic visit for a blood draw (up to 60 days after baseline).

#### Outcome Variables

The primary outcome variable, ease of decision making, was assessed in the fourth website module (see Textbox 1). Participants were asked on a 7-point Likert scale to “tell us how easy or hard it is for you to decide whether or not to get multiplex genetic testing.” Due to the skewed distribution, responses were dichotomized at the midpoint. The second outcome was whether participants had blood drawn for testing.

#### Predictor Variable

The primary predictor variable was the number of website pages viewed for each module. Each page was assigned a value of 0 or 1 depending on whether or not participants viewed that page. The number of pages viewed was then summed overall and within content areas.

#### Mediating Variable

Individuals’ perceptions of the content of the second module (see Textbox 1), which described the health conditions and genes on the test, were rated with respect to trustworthiness, satisfactoriness, helpfulness, and clarity of the information (eg, “I trusted that the information presented was true”). Participants indicated strength of agreement with each item on 7-point Likert scales ranging from “strongly disagree” to “strongly agree.” Four items were reverse coded so that “strongly agree” reflected more positive perceptions for all items. The standardized Cronbach alpha was .76.

#### Covariates

Covariates assessed were based on the Risk Information Seeking and Processing Model [25]. Sociodemographic covariates included gender, age, educational attainment, race, and marital status. Participants also identified which multiplex health conditions ran in their family. A 6-item measure of genetic self-efficacy was adapted from Parrott et al [26]. The items (eg, “You would be able to explain to others how genes affect health”) were answered on 7-point Likert scales ranging from “strongly disagree” to “strongly agree.” Health information seeking was assessed using the item, “In the past 30 days, how often would you say you have looked for information about ways to stay healthy or to feel better?” Responses were dichotomized as daily or weekly versus less than weekly or never. Importance of genetic information was assessed with the item, “How important is it to you to learn more about how your genes affect your chance of getting certain health conditions?” Response choices ranged from 1, not at all important to 7, very important.

### Analysis

Data were analyzed using SAS Version 9.2 for Windows (SAS Institute Inc, Cary, NC, USA). Descriptive statistics were first examined for all variables. Differences by the predictor variable, possible mediating variable, and covariates in the two outcome variables were assessed using chi-square tests. We then tested multivariate logistic regression models to evaluate the association of number of pages viewed with the two outcome variables, employing forward checking and backward elimination methods to determine which covariates to include in the final models [27]. We used a *P* < .20 criterion for inclusion of covariates [28,29]. The potential mediator was tested using the approach of Baron and Kenny [30]. Statistical significance was assessed as *P* < .05.

## Results

### Participant Engagement With Website

Of the 27 possible pages in modules 1 and 2 (Textbox 1), participants viewed, on average, about 8 pages (mean 8.2, standard deviation [SD] 7.2), although the number of pages viewed ranged from 1 to 27. On average, participants viewed about 2.9 of the 4 pages introducing the multiplex test, 2.2 of the 8 pages describing the health conditions on the test, and 3.2 of the 15 pages describing the genes. Over 60% (326/526) of participants viewed the Web page for diabetes, which was the first health condition listed on the website menu. However, less than 25% of participants viewed any of the other health condition pages. Similarly, between 40% and 50% viewed the first gene pages listed on the website menu (for *KCNJ11, CAPN10, PPARG*, and *TCN7L2*), but less than 20% viewed any of the other genes pages. Education was the only significant sociodemographic predictor of the number of Web pages viewed in the multivariate model. Participants with a high school degree or less viewed about 3 1/2 pages fewer, on average, than participants with a college degree or higher (beta = -3.52, *P* < .001), while participants with some college viewed about 2 pages fewer than those with a college degree or higher (beta = -2.10, *P* = .002).

In bivariate analyses, the number of pages viewed was associated with each of the two outcomes (see Table 1). The group of participants who rated their decision to test as easy had looked at more pages within each content area than those who rated the decision as difficult. This difference was about a half page for general test information (*P* = .001) and health condition information (*P* = .02) and about a page for gene information (*P* = .003). Similarly, participants who had blood drawn for the test had viewed more website content than those who declined testing, with differences of about 1 page for information about the test and the health conditions and about 2 pages for information about the genes (*P* < .001).

**Table 1:** Association of website content viewed and ratings of content with decision outcomes (n = 526)

		Ease of Decision		Test Decision	
		Easy Decision (n = 337) Mean (SD)	Difficult Decision (n = 186) Mean (SD)	Received Test (n = 266) Mean (SD)	Did Not Receive Test (n = 260) Mean (SD)
**Pages viewed**					
	General test information (4 pages)	3.0 (1.3)	2.6 (1.4) ^b^	3.2 (1.2)	2.5 (1.4) ^c^
	Health conditions information (8 pages)	2.4 (2.8)	1.8 (2.5) ^a^	2.7 (3.0)	1.6 (2.3) ^c^
	Genes information (15 pages)	3.6 (4.5)	2.5 (4.0) ^b^	4.4 (5.0)	2.0 (3.1) ^c^
**Perceptions of content**					
	Overall perceptions of content	5.5 (1.0)	4.9 (1.0) ^c^	5.5 (1.0)	5.2 (1.0) ^a^
	Trusted information	6.2 (1.1)	5.5 (1.5) ^c^	6.0 (1.2)	5.8 (1.4) ^a^
	Satisfied with information	5.9 (1.2)	5.1 (1.5) ^c^	5.9 (1.2)	5.4 (1.4) ^c^
	Easy to understand	5.7 (1.4)	5.0 (1.6) ^c^	5.6 (1.5)	5.3 (1.6) ^b^
	Able to understand	5.6 (1.7)	5.0 (1.8) ^c^	5.5 (1.8)	5.2 (1.8)
	Helped decision	5.4 (1.7)	4.7 (1.5) ^c^	5.4 (1.6)	4.9 (1.6) ^b^
	Minimal effort to understand	5.3 (1.8)	4.6 (1.9) ^c^	5.2 (1.8)	4.9 (1.9)
	Sufficient information	4.8 (1.9)	4.4 (1.9) ^a^	4.7 (1.9)	4.6 (2.0)

^a^ *P* < .05

^b^ *P* < .01

^c^ *P* < .001

### Participant Ratings of Quality and Usefulness of Information About Health Conditions and Genes 

Participants rated the quality and usefulness of the information about the health conditions and genes positively. These ratings were significantly associated with each of the two outcomes (see Table 1). Participants who rated their decision as easy perceived the website information more positively overall than those who rated their decision as difficult (*P* < .001). This general pattern was also true for individual ratings of trustworthiness of the information (*P* < .001), satisfaction (*P* < .001), ease of understanding (*P* < .001), feeling able to understand the information (*P* < .001), helpfulness of the information (*P* < .001), needing minimal effort to understand the information (*P* < .001), and sufficiency of the information (*P* = .033). Individuals who decided to test rated the website information more positively than those who declined testing (*P <* .001). In the individual ratings, participants who decided to test rated the trustworthiness (*P* = .038), satisfactoriness (*P* < .001), ease of understanding (*P* = .010), and helpfulness of the information (*P* = .001) more positively than those who declined the test.

### Association of Website Usage With Decision-Making

As shown in Table 2, the number of pages viewed was significantly associated with ease of decision-making in multivariate analyses (odds ratio [OR] 1.04, 95% confidence interval [CI] 1.01-1.07). The results of this model showed that for every page viewed, participants were about 4% more likely to describe their decision as easy, on average, controlling for the sociodemographic and psychological covariates. In this model, genetic self-efficacy and involvement with genetic information were significant covariates. Participants with higher genetic self-efficacy (OR 1.27, 95% CI 1.05-1.52) and who placed greater importance on genetic information (OR 1.18, 95% CI 1.03-1.36) were more likely to describe their decision to test or not as easy.

**Table 2:** Prediction of ease of decision making by number of pages viewed in a multivariate logistic regression model (n = 523)

		Odds Ratio	95% Confidence Interval
Number of pages viewed		1.04	(1.01-1.07)
Male gender		0.87	(0.58-1.29)
Age		0.99	(0.94-1.04)
**Education^a^**			
	High school or less	0.81	(0.47-1.39)
	Some college	0.74	(0.49-1.13)
**Race^b^**			
	White	1.00	(0.49-2.05)
	Black	0.58	(0.29-1.19)
Married/partnered		0.96	(0.64-1.45)
Number of conditions with family history		0.94	(0.82-1.07)
Genetic self-efficacy		1.27	(1.05-1.52)
Involvement with genetic information		1.18	(1.03-1.36)

^a^ Comparison category is college degree or higher.

^b^ Comparison category is “other.”

As shown in Table 3, the number of pages viewed also was significantly associated with deciding to test (OR 1.08, 95% CI 1.05-1.11). For every page viewed, participants were about 8% more likely to decide to test, controlling for the sociodemographic and psychological covariates. In this model, education, genetic self-efficacy, and involvement with genetic information were also significant covariates. Individuals with a high school degree or less were about half as likely to be tested compared with those with a college degree or higher (OR 0.51, 95% CI 0.29-0.88). Participants with higher genetic self-efficacy (OR 1.24, 95% CI 1.03-1.50) and those who placed greater importance on genetic information (OR 1.24, 95% CI 1.07-1.44) were more likely to test.

**Table 3:** Prediction of decision to test by number of pages viewed in a multivariate logistic regression model (n = 523)

		Odds Ratio	95% Confidence Interval
Number of pages viewed		1.08	(1.05-1.11)
Male gender		1.26	(0.85-1.89)
Age		1.03	(0.99-1.08)
**Education^a^**			
	High school or less	0.51	(0.29-0.88)
	Some college	1.04	(0.69-1.59)
**Race^b^**			
	White	1.65	(0.84-3.26)
	Black	0.66	(0.33-1.30)
Married/partnered		0.91	(0.60-1.38)
Number of conditions with family history		1.10	(0.97-1.26)
Genetic self efficacy		1.24	(1.03-1.50)
Involvement with genetic information		1.24	(1.07-1.44)

^a^ Comparison category is college degree or higher.

^b^ Comparison category is “other.”

We tested whether perceptions of the quality and usefulness of the information about the health conditions and genes mediated the significant associations of number of pages viewed with decision outcomes. In the first step, we found a low correlation between the number of Web pages viewed and perceptions of the information (*r* = .097). Therefore, we did not proceed to additional steps to test mediation and concluded that perceptions of the website information did not mediate the associations.

## Discussion

This report describes unique data suggesting how individuals respond to Web-based offers of genetic susceptibility tests. This is especially notable because multiplex genetic susceptibility tests currently being offered by many DTC companies have unknown clinical utility. We examined test decisions in a population-based sample where nearly half of participants who visited the website to consider testing ultimately decided not to be tested. This is in contrast to most of the prior genetic testing literature, in which the majority of study participants already had decided to obtain a genetic test [19,20].

Individuals generally had positive perceptions of the quality and usefulness of the website information. Viewing more of the information was associated with finding it easier to decide about testing regardless of whether the individual decided to test or not. Thus, patients found the website helpful in supporting their decision-making—both the decision to test and the decision not to test.

In addition, the results presented here shed light on aspects of the online information that might be most useful in supporting individuals’ decision-making. Participants engaged most with the introductory section that described the test, testing procedures, and what could and could not be learned from the results. This suggests that this information may have been most relevant for their test decisions. In contrast, individuals generally did not delve very deeply into content related to health conditions and gene pages. However, it is noteworthy that participants who described their decision to test as easiest had viewed more of the pages describing the health conditions and genes than those who found it harder to decide. This suggests that more extensive processing about the specifics of the genetic test might have made it easier to decide about testing.

The findings observed here underscore the importance of attending to best communication practices such as layering information in website development. For example, we placed the most important information about the test in the introductory module and then supplemented that with detailed information about each health condition and gene on separate pages. We believe that our observation that participants generally viewed little of the detailed information supports using health literacy best practices. Specifically, the results suggest that information thought to be most essential to individual decision-making be presented first [21,22]. By contrast, a recent analysis of websites offering genetic tests directly to consumers showed that there is wide variability in the content, language, and organizational structure of these sites [18], differences likely to greatly influence their usefulness to consumers.

Despite our attention to health literacy issues (such as reducing technical jargon) in the design of this website, the results showed that educational attainment was the primary predictor of how much information participants viewed. Prior Multiplex Initiative analyses also have shown that educational attainment was associated with whether participants logged onto the website [6]. Supplemental or alternative approaches may be needed to facilitate decision-making among participants with more limited educational attainment or health literacy skills. Individuals with limited health literacy may face substantial challenges in using Web-based information about genomics [16,17], and such consumers might face particular difficulties in making decisions about DTC genetic susceptibility testing [18]. Other factors may also influence the effectiveness of this type of Web-based educational approach, including computer literacy, genetic literacy, and decision-making preferences, all of which are important areas for future research.

Although this population-based study had many strengths, the limitations should also be considered. The observational design did not allow us to examine the effects of individual Web design features or to investigate the effects of the educational material separately from the cognitive characteristics of the participants. For example, some of the observed results may be affected by educational differences in preferences for (or competencies in) reading lengthier text. These are important issues that could be considered in experimental lab-based studies, perhaps with analyses stratified by educational attainment or cognitive characteristics such as information seeking preferences. Similarly, we were not able to drill down to specific information content and decisions about testing. In addition, although we initially drew a population-based sample, participants who logged on to consider genetic testing were more educated and savvier Internet users. Thus, these results may not generalize beyond these early adopters.

The results of this analysis show that consumers perceived a carefully designed website consistent with best practices in communication to be helpful in deciding about genetic susceptibility testing. Critical next steps in this area will be to examine individuals’ understanding and interpretation of such website information and how it affects responses to test feedback. For example, is better understanding of the limitations of genetic susceptibility testing associated with more accurate interpretations of test feedback?

As genomic discovery advances, Web-based delivery likely will continue and expand as an avenue for education and decision support regarding genetic testing. These results suggest that individuals perceive Web-based tools designed based on evidence-based communication approaches as supporting decision-making about genetic testing in some target groups. However, continued research is needed to ensure that these tools or other appropriate decision support approaches are available to all groups including those having lower literacy.

## Abbreviations

CIDR: Center for Inherited Disease Research
DTC: direct-to-consumer
NHGRI: National Human Genome Research Institute

## References

[1] McBride CM, Alford SH, Reid RJ, Larson EB, Baxevanis AD, Brody LC (2008). Putting science over supposition in the arena of personalized genomics. Nat Genet.

[2] Collins FS, Green ED, Guttmacher AE, Guyer MS (2003). A vision for the future of genomics research. Nature.

[3] Topol EJ, Murray SS, Frazer KA (2007). The genomics gold rush. JAMA.

[4] Katsanis SH, Javitt G, Hudson K (2008). Public health. A case study of personalized medicine. Science.

[5] McBride CM, Alford SH, Reid RJ, Larson EB, Baxevanis AD, Brody LC (2009). Characteristics of users of online personalized genomic risk assessments: implications for physician-patient interactions. Genet Med.

[6] Hensley Alford S, McBride CM, Reid RJ, Larson EB, Baxevanis AD, Brody LC (2010). Participation in genetic testing research varies by social group. Public Health Genomics.

[7] Hunter DJ, Khoury MJ, Drazen JM (2008). Letting the genome out of the bottle--will we get our wish?. N Engl J Med.

[8] Gollust SE, Hull SC, Wilfond BS (2002). Limitations of direct-to-consumer advertising for clinical genetic testing. JAMA.

[9] Wasson K, Cook ED, Helzlsouer K (2006). Direct-to-consumer online genetic testing and the four principles: an analysis of the ethical issues. Ethics Med.

[10] Ensenauer RE, Michels VV, Reinke SS (2005). Genetic testing: practical, ethical, and counseling considerations. Mayo Clin Proc.

[11] Geransar R, Einsiedel E (2008). Evaluating online direct-to-consumer marketing of genetic tests: informed choices or buyers beware?. Genet Test.

[12] Goddard KA, Robitaille J, Dowling NF, Parrado AR, Fishman J, Bradley LA, Moore CA, Khoury MJ (2009). Health-related direct-to-consumer genetic tests: a public health assessment and analysis of practices related to Internet-based tests for risk of thrombosis. Public Health Genomics.

[13] Horrigan J (2006). Home Broadband Adoption 2006.

[14] Sanderson SC, O'Neill SC, White DB, Bepler G, Bastian L, Lipkus IM, McBride CM (2009). Responses to online GSTM1 genetic test results among smokers related to patients with lung cancer: a pilot study. Cancer Epidemiol Biomarkers Prev.

[15] Birru MS, Monaco VM, Charles L, Drew H, Njie V, Bierria T, Detlefsen E, Steinman RA (2004). Internet usage by low-literacy adults seeking health information: an observational analysis. J Med Internet Res.

[16] Kaphingst KA, Zanfini CJ, Emmons KM (2006). Accessibility of web sites containing colorectal cancer information to adults with limited literacy (United States). Cancer Causes Control.

[17] Johnson JD, Case DO, Andrews JE, Allard SL (2005). Genomics--the perfect information-seeking research problem. J Health Commun.

[18] Lachance CR, Erby LA, Ford BM, Allen VC Jr, Kaphingst KA (2010). Informational content, literacy demands, and usability of websites offering health-related genetic tests directly to consumers. Genet Med.

[19] Ropka ME, Wenzel J, Phillips EK, Siadaty M, Philbrick JT (2006). Uptake rates for breast cancer genetic testing: a systematic review. Cancer Epidemiol Biomarkers Prev.

[20] Hadley DW, Jenkins J, Dimond E, Nakahara K, Grogan L, Liewehr DJ, Steinberg SM, Kirsch I (2003). Genetic counseling and testing in families with hereditary nonpolyposis colorectal cancer. Arch Intern Med.

[21] Doak C, Doak L, Root J (1996). Teaching Patients with Low Literacy Skills, 2nd edition.

[22] US Department of Health and Human Services (2006). Usability.gov.

[23] Lipkus IM, Hollands JG (1999). The visual communication of risk. J Natl Cancer Inst Monogr.

[24] Fagerlin A, Ubel PA, Smith DM, Zikmund-Fisher BJ (2007). Making numbers matter: present and future research in risk communication. Am J Health Behav.

[25] Griffin RJ, Dunwoody S, Neuwirth K (1999). Proposed model of the relationship of risk information seeking and processing to the development of preventive behaviors. Environ Res.

[26] Parrott R, Silk K, Raup Krieger J, Harris T, Condit C (2004). Behavioral health outcomes associated with religious faith and media exposure about human genetics. Health Commun.

[27] Hosmer D, Lemeshow S (2000). Applied Logistic Regression.

[28] Maldonado G, Greenland S (1993). Simulation study of confounder-selection strategies. Am J Epidemiol.

[29] Budtz-Jørgensen E, Keiding N, Grandjean P, Weihe P (2007). Confounder selection in environmental epidemiology: assessment of health effects of prenatal mercury exposure. Ann Epidemiol.

[30] Baron RM, Kenny DA (1986). The moderator-mediator variable distinction in social psychological research: conceptual, strategic, and statistical considerations. J Pers Soc Psychol.

